# 1-Do­decyl­indoline-2,3-dione

**DOI:** 10.1107/S1600536814001792

**Published:** 2014-01-31

**Authors:** Fatima-Zahrae Qachchachi, Fouad Ouazzani Chahdi, Houria Misbahi, Michael Bodensteiner, Lahcen El Ammari

**Affiliations:** a Laboratoire de Chimie Organique Appliquée, Université Sidi Mohamed Ben Abdallah, Faculté des Sciences et Techniques, Route d’Immouzzer, BP 2202 Fès, Morocco; b Institut National des Plantes Médicinales et Aromatiques, Université Sidi Mohamed Ben Abdallah, Fès, Morocco; c X-Ray Structure Analysis, University of Regensburg, D-93053 Regensburg, Germany; d Laboratoire de Chimie du Solide Appliquée, Faculté des Sciences, Université Mohammed V-Agdal, Avenue Ibn Battouta, BP 1014, Rabat, Morocco

## Abstract

The structure of the title compound, C_20_H_29_NO_2_, is isotypic to that of its homologue 1-octylindoline-2,3-dione. The indoline ring and the two carbonyl-group O atoms are approximately coplanar, the largest deviation from the mean plane being 0.0760 (10) Å. The mean plane through the fused-ring system is nearly perpendicular to the mean plane passing through the 1-dodecyl chain [dihedral angle = 77.69 (5)°]. All C atoms of the dodecyl group are in an anti­periplanar arrangement. In the crystal, mol­ecules are linked by C—H⋯O hydrogen bonds, forming a three-dimensional network.

## Related literature   

For biological activity of indoline derivatives, see: Bhrigu *et al.* (2010 ▶); Malhotra *et al.* (2011 ▶); Da Silva *et al.* (2001 ▶); Ramachandran (2011 ▶); Smitha *et al.* (2008 ▶). For similar compounds see: Qachchachi *et al.* (2013 ▶).
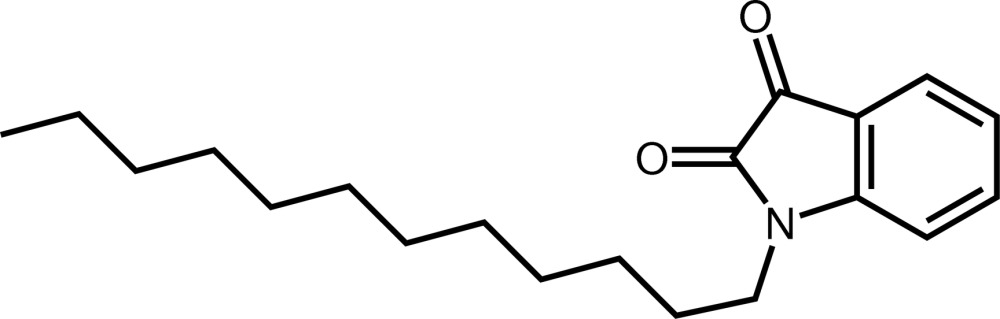


## Experimental   

### 

#### Crystal data  

C_20_H_29_NO_2_
*M* *_r_* = 315.44
Monoclinic, 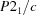

*a* = 25.2013 (7) Å
*b* = 4.66818 (9) Å
*c* = 15.7013 (4) Å
β = 104.926 (3)°
*V* = 1784.84 (7) Å^3^
*Z* = 4
Cu *K*α radiation
μ = 0.58 mm^−1^
*T* = 123 K
0.12 × 0.11 × 0.04 mm

#### Data collection  

Oxford Diffraction SuperNova (single source at offset, Atlas) diffractometer
Absorption correction: analytical [*CrysAlis PRO* (Oxford Diffraction, 2012 ▶); analytical numeric absorption correction using a multi-faceted crystal model (Clark & Reid, 1995 ▶)] *T*_min_ = 0.942, *T*_max_ = 0.979
12640 measured reflections
3493 independent reflections
3039 reflections with *I* > 2σ(*I*)
*R*_int_ = 0.021

#### Refinement  

*R*[*F* ^2^ > 2σ(*F* ^2^)] = 0.035
*wR*(*F* ^2^) = 0.100
*S* = 1.02
3493 reflections
208 parameters
H-atom parameters constrained
Δρ_max_ = 0.29 e Å^−3^
Δρ_min_ = −0.20 e Å^−3^

### 

Data collection: *CrysAlis PRO* (Oxford Diffraction, 2012 ▶); cell refinement: *CrysAlis PRO*; data reduction: *CrysAlis PRO*; program(s) used to solve structure: *SHELXS97* (Sheldrick, 2008 ▶); program(s) used to refine structure: *SHELXL97* (Sheldrick, 2008 ▶); molecular graphics: *ORTEP-3 for Windows* (Farrugia, 2012 ▶); software used to prepare material for publication: *WinGX* (Farrugia, 2012 ▶) and *publCIF* (Westrip, 2010 ▶).

## Supplementary Material

Crystal structure: contains datablock(s) I. DOI: 10.1107/S1600536814001792/rz5103sup1.cif

Structure factors: contains datablock(s) I. DOI: 10.1107/S1600536814001792/rz5103Isup2.hkl

Supporting information file. DOI: 10.1107/S1600536814001792/rz5103Isup3.cml

CCDC reference:

Additional supporting information:  crystallographic information; 3D view; checkCIF report

## Figures and Tables

**Table 1:** Hydrogen-bond geometry (Å, °)

*D*—H⋯*A*	*D*—H	H⋯*A*	*D*⋯*A*	*D*—H⋯*A*
C6—H6⋯O1^i^	0.95	2.47	3.1423 (14)	127
C6—H6⋯O2^ii^	0.95	2.55	3.2360 (13)	130
C8—H8⋯O2^iii^	0.95	2.52	3.4598 (13)	169

Symmetry codes: (i) 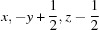; (ii) 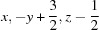; (iii) 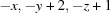.

**Fig. 1.**
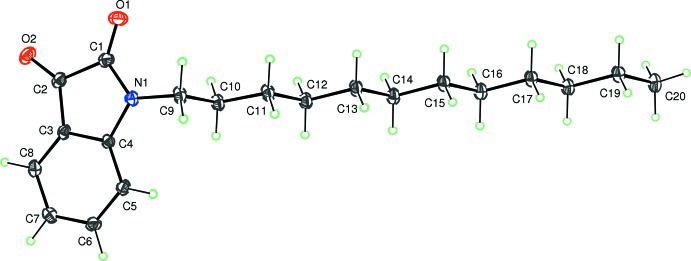
Molecular plot of the title compound with displacement ellipsoids drawn at the 50% probability level. H atoms are represented as small circles.

**Fig. 2.**
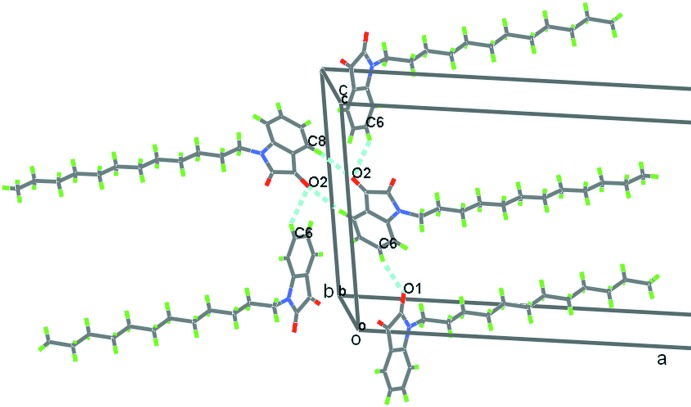
Intermolecular hydrogen interactions in the title compound. Hydrogen bonds are shown as dashed lines.

## supplementary crystallographic information

### 1. Comment

Isatin (1*H*-indoline-2,3-dione) and derivatives possess a broad range of biological and pharmacological properties and are widely used as starting materials for the synthesis of heterocyclic compounds and as substrates for drug synthesis relevant to application as insecticides and fungicides. These compounds find applications also in a broad range of therapies as anticancer drugs, antibiotics and antidepressants (Bhrigu *et al.*, 2010; Malhotra *et al.*, 2011; Da Silva *et al.*, 2001; Ramachandran, 2011; Smitha *et al.*, 2008). In our work, we are interested in developing a new isatin derivative with the addition of an alkyl halide to explore other potential applications.

The molecule of title compound is build up from a fused five- and six-membered ring system linked to a 1-dodecyl chain and to two ketone O atoms as shown in Fig. 1. The indoline ring and the two carbonyl-group O atoms are nearly coplanar, the largest deviation from the mean plane being 0.0760 (10) Å for atom O1. The plane of the fused ring system is nearly perpendicular to the mean plane passing through the 1-dodecyl chain as indicated by the dihedral angle of 77.69 (5)°. The dodecyl substituent has all carbon atoms in an antiperiplanar conformation. The structure of the title compound is similar to that of its homologue 1-octylindoline-2,3-dione (Qachchachi *et al.*, 2013).

In the crystal, the molecules are linked by C6–H6···O1, C6–H6···O2 and C8–H8···O2 hydrogen bonds in the way to build a three-dimensional network as shown in Fig. 2 and Table 2.

### 2. Experimental

To a solution of isatin (0.5 g, 3.4 mmol) dissolved in DMF (30 ml) was added potassium carbonate (0.61 g, 4.4 mmol), a catalytic quantity of tetra-n-butylammonium bromide (0.1 g, 0.4 mmol) and 1-bromododecane (0.9 ml, 3.7 mmol). The mixture was stirred for 48 h and the reaction monitored by thin layer chromatography. The mixture was filtered and the solvent removed under vacuum. The solid obtained was recrystallized from ethanol to afford the title compound as orange crystals in 86% yield (m. p. 321 K).

### 3. Refinement

All H atoms could be located in a difference Fourier map. However, they were placed in calculated positions with C—H = 0.95 Å (aromatic), C—H = 0.99 Å (methylene) and C—H = 0.97 Å (methyl) and refined as riding on their parent atoms with *U*_iso_(H) = 1.2 *U*_eq_ (C) or *U*_iso_(H) = 1.5 *U*_eq_(C) for methyl H atoms.

### Crystal data

**Table:**

C_20_H_29_NO_2_	*F*(000) = 688
*M**_r_* = 315.44	*D*_x_ = 1.174 Mg m^−^^3^
Monoclinic, *P*2_1_/*c*	Cu *K*α radiation, λ = 1.54184 Å
Hall symbol: -P 2ybc	Cell parameters from 6237 reflections
*a* = 25.2013 (7) Å	θ = 5.4–73.8°
*b* = 4.66818 (9) Å	µ = 0.58 mm^−^^1^
*c* = 15.7013 (4) Å	*T* = 123 K
β = 104.926 (3)°	Plate, clear light orange
*V* = 1784.84 (7) Å^3^	0.12 × 0.11 × 0.04 mm
*Z* = 4	

### Data collection

**Table:**

Oxford Diffraction SuperNova (single source at offset, Atlas) diffractometer	3493 independent reflections
Radiation source: SuperNova (Cu) X-ray Source	3039 reflections with *I* > 2σ(*I*)
Mirror monochromator	*R*_int_ = 0.021
Detector resolution: 10.3546 pixels mm^-1^	θ_max_ = 73.6°, θ_min_ = 3.6°
ω scans	*h* = −31→30
Absorption correction: analytical [*CrysAlis PRO* (Oxford Diffraction, 2012); analytical numeric absorption correction using a multi-faceted crystal model (Clark & Reid, 1995)]	*k* = −5→5
*T*_min_ = 0.942, *T*_max_ = 0.979	*l* = −19→18
12640 measured reflections	

### Refinement

**Table:**

Refinement on *F*^2^	Primary atom site location: structure-invariant direct methods
Least-squares matrix: full	Secondary atom site location: difference Fourier map
*R*[*F*^2^ > 2σ(*F*^2^)] = 0.035	Hydrogen site location: inferred from neighbouring sites
*wR*(*F*^2^) = 0.100	H-atom parameters constrained
*S* = 1.02	*w* = 1/[σ^2^(*F*_o_^2^) + (0.0528*P*)^2^ + 0.4135*P*] where *P* = (*F*_o_^2^ + 2*F*_c_^2^)/3
3493 reflections	(Δ/σ)_max_ = 0.001
208 parameters	Δρ_max_ = 0.29 e Å^−^^3^
0 restraints	Δρ_min_ = −0.20 e Å^−^^3^

### Special details

**Table:**

Geometry. All e.s.d.'s (except the e.s.d. in the dihedral angle between two l.s. planes) are estimated using the full covariance matrix. The cell e.s.d.'s are taken into account individually in the estimation of e.s.d.'s in distances, angles and torsion angles; correlations between e.s.d.'s in cell parameters are only used when they are defined by crystal symmetry. An approximate (isotropic) treatment of cell e.s.d.'s is used for estimating e.s.d.'s involving l.s. planes.
Refinement. Refinement of *F*^2^ against all reflections. The weighted *R*-factor *wR* and goodness of fit *S* are based on *F*^2^, conventional *R*-factors *R* are based on *F*, with *F* set to zero for negative *F*^2^. The threshold expression of *F*^2^ > σ(*F*^2^) is used only for calculating *R*-factors(gt) *etc*. and is not relevant to the choice of reflections for refinement. *R*-factors based on *F*^2^ are statistically about twice as large as those based on *F*, and *R*- factors based on all data will be even larger.

### Fractional atomic coordinates and isotropic or equivalent isotropic displacement parameters (Å^2^)

**Table:**

	*x*	*y*	*z*	*U*_iso_*/*U*_eq_	
C1	0.11717 (4)	0.3582 (2)	0.56405 (7)	0.0263 (2)	
C2	0.07145 (4)	0.5831 (2)	0.52742 (7)	0.0256 (2)	
C3	0.07308 (4)	0.6313 (2)	0.43624 (7)	0.0221 (2)	
C4	0.11376 (4)	0.4511 (2)	0.41950 (7)	0.0207 (2)	
C5	0.12551 (4)	0.4485 (2)	0.33845 (7)	0.0243 (2)	
H5	0.1527	0.3249	0.3268	0.029*	
C6	0.09572 (4)	0.6350 (2)	0.27432 (7)	0.0264 (2)	
H6	0.1030	0.6382	0.2179	0.032*	
C7	0.05561 (4)	0.8162 (2)	0.29048 (7)	0.0280 (2)	
H7	0.0363	0.9419	0.2455	0.034*	
C8	0.04351 (4)	0.8149 (2)	0.37185 (7)	0.0258 (2)	
H8	0.0158	0.9361	0.3831	0.031*	
C9	0.18532 (4)	0.0978 (2)	0.50360 (8)	0.0262 (2)	
H9A	0.1802	−0.0161	0.4489	0.031*	
H9B	0.1870	−0.0370	0.5529	0.031*	
C10	0.23949 (4)	0.2615 (2)	0.52023 (8)	0.0264 (2)	
H10A	0.2396	0.3770	0.4674	0.032*	
H10B	0.2421	0.3951	0.5701	0.032*	
C11	0.28944 (4)	0.0655 (2)	0.54105 (8)	0.0261 (2)	
H11A	0.2866	−0.0697	0.4915	0.031*	
H11B	0.2896	−0.0483	0.5943	0.031*	
C12	0.34340 (4)	0.2303 (2)	0.55654 (8)	0.0269 (2)	
H12A	0.3441	0.3339	0.5018	0.032*	
H12B	0.3448	0.3748	0.6032	0.032*	
C13	0.39431 (4)	0.0414 (3)	0.58348 (8)	0.0275 (2)	
H13A	0.3930	−0.1032	0.5369	0.033*	
H13B	0.3938	−0.0620	0.6383	0.033*	
C14	0.44783 (4)	0.2094 (3)	0.59858 (8)	0.0283 (3)	
H14A	0.4485	0.3105	0.5434	0.034*	
H14B	0.4487	0.3563	0.6444	0.034*	
C15	0.49914 (4)	0.0237 (3)	0.62696 (8)	0.0285 (3)	
H15A	0.4984	−0.1232	0.5812	0.034*	
H15B	0.4986	−0.0771	0.6822	0.034*	
C16	0.55241 (4)	0.1948 (3)	0.64174 (8)	0.0290 (3)	
H16A	0.5528	0.2957	0.5865	0.035*	
H16B	0.5531	0.3416	0.6875	0.035*	
C17	0.60399 (4)	0.0110 (3)	0.67010 (8)	0.0290 (3)	
H17A	0.6033	−0.1358	0.6243	0.035*	
H17B	0.6036	−0.0900	0.7254	0.035*	
C18	0.65705 (4)	0.1830 (3)	0.68478 (8)	0.0287 (3)	
H18A	0.6574	0.2838	0.6294	0.034*	
H18B	0.6576	0.3302	0.7304	0.034*	
C19	0.70879 (4)	0.0017 (3)	0.71327 (8)	0.0319 (3)	
H19A	0.7083	−0.1461	0.6678	0.038*	
H19B	0.7087	−0.0981	0.7689	0.038*	
C20	0.76140 (5)	0.1765 (3)	0.72716 (9)	0.0383 (3)	
H20A	0.7626	0.3204	0.7731	0.046*	
H20B	0.7622	0.2724	0.6720	0.046*	
H20C	0.7932	0.0491	0.7453	0.046*	
N1	0.13859 (4)	0.2877 (2)	0.49531 (6)	0.0237 (2)	
O1	0.13183 (4)	0.2676 (2)	0.63893 (5)	0.0368 (2)	
O2	0.04303 (3)	0.6876 (2)	0.57083 (5)	0.0350 (2)	

### Atomic displacement parameters (Å^2^)

**Table:**

	*U*^11^	*U*^22^	*U*^33^	*U*^12^	*U*^13^	*U*^23^
C1	0.0228 (5)	0.0325 (6)	0.0234 (5)	−0.0083 (4)	0.0058 (4)	−0.0017 (5)
C2	0.0203 (5)	0.0317 (6)	0.0250 (5)	−0.0069 (4)	0.0063 (4)	−0.0076 (5)
C3	0.0169 (4)	0.0255 (5)	0.0240 (5)	−0.0034 (4)	0.0056 (4)	−0.0056 (4)
C4	0.0168 (4)	0.0231 (5)	0.0212 (5)	−0.0028 (4)	0.0029 (4)	−0.0021 (4)
C5	0.0201 (5)	0.0290 (5)	0.0248 (5)	0.0006 (4)	0.0077 (4)	−0.0035 (4)
C6	0.0262 (5)	0.0324 (6)	0.0206 (5)	−0.0028 (5)	0.0063 (4)	−0.0013 (4)
C7	0.0248 (5)	0.0290 (6)	0.0274 (6)	0.0008 (4)	0.0017 (4)	0.0018 (5)
C8	0.0192 (5)	0.0263 (5)	0.0308 (6)	0.0010 (4)	0.0044 (4)	−0.0040 (5)
C9	0.0192 (5)	0.0256 (5)	0.0320 (6)	0.0005 (4)	0.0034 (4)	0.0025 (5)
C10	0.0192 (5)	0.0257 (5)	0.0322 (6)	−0.0005 (4)	0.0031 (4)	0.0015 (5)
C11	0.0188 (5)	0.0277 (5)	0.0297 (6)	0.0002 (4)	0.0025 (4)	0.0019 (5)
C12	0.0193 (5)	0.0294 (6)	0.0308 (6)	−0.0003 (4)	0.0042 (4)	0.0016 (5)
C13	0.0188 (5)	0.0316 (6)	0.0308 (6)	0.0004 (4)	0.0038 (4)	0.0014 (5)
C14	0.0189 (5)	0.0336 (6)	0.0315 (6)	0.0002 (4)	0.0046 (4)	0.0015 (5)
C15	0.0188 (5)	0.0342 (6)	0.0315 (6)	0.0005 (4)	0.0043 (4)	0.0023 (5)
C16	0.0191 (5)	0.0345 (6)	0.0323 (6)	0.0000 (4)	0.0048 (4)	0.0004 (5)
C17	0.0194 (5)	0.0357 (6)	0.0308 (6)	0.0012 (5)	0.0047 (4)	0.0034 (5)
C18	0.0194 (5)	0.0355 (6)	0.0300 (6)	0.0003 (4)	0.0041 (4)	0.0005 (5)
C19	0.0217 (5)	0.0403 (6)	0.0326 (6)	0.0029 (5)	0.0052 (5)	0.0050 (5)
C20	0.0193 (5)	0.0507 (8)	0.0428 (7)	0.0010 (5)	0.0044 (5)	0.0007 (6)
N1	0.0187 (4)	0.0286 (5)	0.0232 (5)	−0.0001 (4)	0.0042 (3)	0.0013 (4)
O1	0.0369 (5)	0.0487 (5)	0.0239 (4)	−0.0068 (4)	0.0060 (3)	0.0062 (4)
O2	0.0287 (4)	0.0505 (5)	0.0288 (4)	−0.0021 (4)	0.0127 (3)	−0.0127 (4)

### Geometric parameters (Å, º)

**Table:**

C1—O1	1.2142 (14)	C12—H12A	0.9900
C1—N1	1.3656 (14)	C12—H12B	0.9900
C1—C2	1.5552 (16)	C13—C14	1.5252 (14)
C2—O2	1.2113 (13)	C13—H13A	0.9900
C2—C3	1.4601 (14)	C13—H13B	0.9900
C3—C8	1.3869 (16)	C14—C15	1.5251 (15)
C3—C4	1.4027 (14)	C14—H14A	0.9900
C4—C5	1.3789 (14)	C14—H14B	0.9900
C4—N1	1.4163 (14)	C15—C16	1.5275 (14)
C5—C6	1.3950 (16)	C15—H15A	0.9900
C5—H5	0.9500	C15—H15B	0.9900
C6—C7	1.3909 (16)	C16—C17	1.5250 (15)
C6—H6	0.9500	C16—H16A	0.9900
C7—C8	1.3878 (15)	C16—H16B	0.9900
C7—H7	0.9500	C17—C18	1.5257 (15)
C8—H8	0.9500	C17—H17A	0.9900
C9—N1	1.4528 (13)	C17—H17B	0.9900
C9—C10	1.5270 (14)	C18—C19	1.5220 (15)
C9—H9A	0.9900	C18—H18A	0.9900
C9—H9B	0.9900	C18—H18B	0.9900
C10—C11	1.5222 (14)	C19—C20	1.5239 (16)
C10—H10A	0.9900	C19—H19A	0.9900
C10—H10B	0.9900	C19—H19B	0.9900
C11—C12	1.5265 (14)	C20—H20A	0.9800
C11—H11A	0.9900	C20—H20B	0.9800
C11—H11B	0.9900	C20—H20C	0.9800
C12—C13	1.5238 (14)		
			
O1—C1—N1	126.70 (11)	C14—C13—H13A	108.9
O1—C1—C2	127.24 (10)	C12—C13—H13B	108.9
N1—C1—C2	106.04 (9)	C14—C13—H13B	108.9
O2—C2—C3	131.26 (11)	H13A—C13—H13B	107.8
O2—C2—C1	123.57 (10)	C15—C14—C13	113.73 (10)
C3—C2—C1	105.16 (8)	C15—C14—H14A	108.8
C8—C3—C4	121.03 (10)	C13—C14—H14A	108.8
C8—C3—C2	131.64 (10)	C15—C14—H14B	108.8
C4—C3—C2	107.32 (9)	C13—C14—H14B	108.8
C5—C4—C3	121.28 (10)	H14A—C14—H14B	107.7
C5—C4—N1	128.13 (9)	C14—C15—C16	113.14 (10)
C3—C4—N1	110.58 (9)	C14—C15—H15A	109.0
C4—C5—C6	117.21 (9)	C16—C15—H15A	109.0
C4—C5—H5	121.4	C14—C15—H15B	109.0
C6—C5—H5	121.4	C16—C15—H15B	109.0
C7—C6—C5	121.95 (10)	H15A—C15—H15B	107.8
C7—C6—H6	119.0	C17—C16—C15	113.57 (10)
C5—C6—H6	119.0	C17—C16—H16A	108.9
C8—C7—C6	120.51 (10)	C15—C16—H16A	108.9
C8—C7—H7	119.7	C17—C16—H16B	108.9
C6—C7—H7	119.7	C15—C16—H16B	108.9
C3—C8—C7	118.01 (10)	H16A—C16—H16B	107.7
C3—C8—H8	121.0	C16—C17—C18	113.33 (10)
C7—C8—H8	121.0	C16—C17—H17A	108.9
N1—C9—C10	112.22 (9)	C18—C17—H17A	108.9
N1—C9—H9A	109.2	C16—C17—H17B	108.9
C10—C9—H9A	109.2	C18—C17—H17B	108.9
N1—C9—H9B	109.2	H17A—C17—H17B	107.7
C10—C9—H9B	109.2	C19—C18—C17	113.75 (10)
H9A—C9—H9B	107.9	C19—C18—H18A	108.8
C11—C10—C9	112.92 (9)	C17—C18—H18A	108.8
C11—C10—H10A	109.0	C19—C18—H18B	108.8
C9—C10—H10A	109.0	C17—C18—H18B	108.8
C11—C10—H10B	109.0	H18A—C18—H18B	107.7
C9—C10—H10B	109.0	C18—C19—C20	113.08 (11)
H10A—C10—H10B	107.8	C18—C19—H19A	109.0
C10—C11—C12	112.63 (9)	C20—C19—H19A	109.0
C10—C11—H11A	109.1	C18—C19—H19B	109.0
C12—C11—H11A	109.1	C20—C19—H19B	109.0
C10—C11—H11B	109.1	H19A—C19—H19B	107.8
C12—C11—H11B	109.1	C19—C20—H20A	109.5
H11A—C11—H11B	107.8	C19—C20—H20B	109.5
C13—C12—C11	113.86 (9)	H20A—C20—H20B	109.5
C13—C12—H12A	108.8	C19—C20—H20C	109.5
C11—C12—H12A	108.8	H20A—C20—H20C	109.5
C13—C12—H12B	108.8	H20B—C20—H20C	109.5
C11—C12—H12B	108.8	C1—N1—C4	110.82 (9)
H12A—C12—H12B	107.7	C1—N1—C9	123.54 (9)
C12—C13—C14	113.16 (9)	C4—N1—C9	125.19 (9)
C12—C13—H13A	108.9		

### Hydrogen-bond geometry (Å, º)

**Table:**

*D*—H···*A*	*D*—H	H···*A*	*D*···*A*	*D*—H···*A*
C6—H6···O1^i^	0.95	2.47	3.1423 (14)	127
C6—H6···O2^ii^	0.95	2.55	3.2360 (13)	130
C8—H8···O2^iii^	0.95	2.52	3.4598 (13)	169

Symmetry codes: (i) *x*, −*y*+1/2, *z*−1/2; (ii) *x*, −*y*+3/2, *z*−1/2; (iii) −*x*, −*y*+2, −*z*+1.
